# A Word of Advice on the Teeth

**Published:** 1840-07

**Authors:** C. S. Brewster

**Affiliations:** PARIS, FRANCE.


The following comes to us in the form of a circular, intended by Mr. Brewster to lie upon the table of his waiting room for the benefit of his patients; and as it is the kind of advice that not only all patients, but, if followed out, that all dentists would be benefitted by, and as we are well apprised of Mr. B's well earned reputation, we feel happy in inserting it, although it is done without his knowledge or permission.

### A WORD OF ADVICE ON THE TEETH.

[ BY C. S. BREWSTER,—PARIS, FRANCE. ]

Incipient teeth may be extracted too soon or left too long; if they loosen themselves by the absorption of their fangs and allow the permanent to take their proper place, all will be well; but if the first teeth remain firm and the second on their appearance require room; do not delay to give them place by removing the adjoining of the first dentition. The permanent teeth must have sufficient room or irregularity and decay will ensue. At the age of five years, give your children tooth brushes and teach them to cleanse the mouth and teeth morning and evening, especially on going to bed. At that age, powder is not requisite but cleanliness is necessary at all ages. Food, fruit, etc., etc., left in the mouth and between the teeth during sleep are the principal causes of their decay. Give your own teeth the same attention, tooth-picks are indispensable for their preservation ;* let your brush be as hard and your water as cool as you can comfortably support. Twice or thrice a week, use dentrifice containing no deleterious substance ; carefully avoid powders and elixirs in which there is any acid ; suffer no acid, whether taken in food or drink, to remain in the mouth; keep your teeth clean, content yourself with their natural colour. Any effort to give them an unnatural whiteness will injure them. Your dentist possesses your confidence or you will choose another, therefore go to him at least twice a year ; allow him to do what he thinks best ; pay him and don't stop to waste his time with long and useless relations of past calamities ; his time is precious, other persons who value their teeth as you value yours, are waiting their turn for his aid. Recollect that early and careful attention to your teeth, cleanliness of mouth, temperance in living and abstinence from acids, are the best maxims for the beauty and preservation of your teeth.

* The only tooth-pick that is at once safe, convenient and effectual, is untwisted, or floss silk well waxed. This will pass readily between the teeth how close soever they may be, which is not true of any other substance that can be obtained with equal convenience. It will moreover remove all impurities from between the teeth and under the gums, and keep the enamel highly polished if used with sufficient frequency and with proper care. Let any person try it, who doubts the propriety of this recommendation.—S. BROWN.

